# Effects of a Self-Management Program on Adults with Stroke: A Quasi-Experimental Study

**DOI:** 10.3390/healthcare13050495

**Published:** 2025-02-25

**Authors:** Carla M. Pereira, Daniela Branco, Dina Salvador, Teresa L. Dias, Daniel Carvalho, Mara Matos, Sandra Rodrigues, José M. Calheiros, António Manuel Marques, Fiona Jones

**Affiliations:** 1 School of Health, Polytechnic University of Setúbal, 2910-761 Setúbal, Portugal; 220537004@estudantes.ips.pt (D.B.); teresa.dias@ess.ips.pt (T.L.D.); sandra.rodrigues@ess.ips.pt (S.R.); antonio.marques@ess.ips.pt (A.M.M.); 2 Comprehensive Health Research Centre (CHRC), NOVA University of Lisbon, 1150-082 Lisbon, Portugal; 3 Setúbal School of Technology, Polytechnic University of Setúbal, 2910-761 Setúbal, Portugal; dina.salvador@estsetubal.ips.pt; 4 Local Health Unit Litoral Alentejano (ULSLA), 7540-230 Santiago do Cacém, Portugal; daniel.carvalho@ulsla.min-saude.pt; 5 NOVA Clinical Research Unit, Nova Medical School, 1150-190 Lisbon, Portugal; maramatos@nms.unl.pt; 6 Institute for Research, Innovation and Development (FP-I3ID), University Fernando Pessoa, 4249-004 Porto, Portugal; jcalheiros@ufp.edu.pt; 7 Population Health Research Institute, St George’s, University of London, London SW17 ORE, UK; fjones@sgul.ac.uk

**Keywords:** stroke, self-management, self-efficacy, quality of life, patient participation

## Abstract

Background: Stroke is a leading cause of death and disability, underscoring the importance of effective self-management programs to improve the quality of life for survivors. Objectives: This study investigates the impact of the ComVida (Bridges-PT) self-management program on self-efficacy, physical function, health-related quality of life, and emotional state of stroke survivors in Portugal. Methods: A quasi-experimental study was conducted with 28 participants from hospital and community settings. The ComVida program, combining personalized rehabilitation sessions and the use of a self-management workbook was implemented. Assessments were conducted at baseline, 6 weeks, and 12 weeks using the Stroke Self-Efficacy Questionnaire (SSEQ), the Stroke Impact Scale (SIS-16), Hospital Anxiety and Depression Scale (HADS), and Short Form Questionnaire-12 (SF-12v2). Results: Significant improvements were observed in self-efficacy, physical function, emotional state, and health-related quality of life over the study period. The SSEQ scores increased from 23.3 at baseline to 33.3 at 12 weeks, while SIS-16 scores improved from 47.5 to 67.2. Anxiety and depression levels, measured by HADS, also showed significant reductions, as did health-related quality of life, evaluated by the SF-12v2. Conclusions: The results suggest that the program may enhance self-efficacy, physical function, and emotional well-being in stroke survivors, highlighting its potential as a valuable component of post-stroke care in Portugal.

## 1. Introduction

Self-management programs are acknowledged as an effective approach for assisting individuals with long-term and neurological conditions [1,2], such as stroke, in managing their daily lives [3]. Given the significant global burden of stroke, the need for effective self-management programs is critical.

Stroke is one of the leading causes of death and disability worldwide, affecting millions of individuals annually [4]. In 2021, stroke, ischemic heart disease, and neonatal disorders continued to be among the leading causes of Disability-Adjusted Life Years (DALYs) globally [5]. With over one hundred one million people worldwide living with the aftermath of a stroke and one in four people over the age of 25 expected to experience a stroke in their lifetime [4], the relevance of self-management programs cannot be overstated. In Portugal, the impact of stroke is even more pronounced, as it is the primary cause of death and disability among the elderly [6]. Beyond human losses, stroke imposes considerable direct and indirect impacts on patients, caregivers, and families [7]. The sequelae can range from motor and cognitive impairments to alterations in speech, vision, and functional autonomy, profoundly affecting survivors and their caregivers. Given these significant impacts, focusing on self-management after a stroke is highly relevant.

International stroke guidelines [3,8,9] and the World Stroke Organization [10] recommend that self-management support be a fundamental part of stroke rehabilitation. Systematic reviews have shown that such interventions can lead to improvements in physical and functional domains, dependency, quality of life, self-efficacy, and reductions in hospital readmission rates and healthcare utilization [11,12,13,14]. These programs aim to support stroke survivors in navigating the challenges of their condition and leading to better long-term health and well-being by empowering people to take control of their health [11,12]. Previous reviews support the use of the following self-management strategies: (i) involving people in decision-making, developing care plans in partnership, goal setting, and follow-up; (ii) emphasizing problem-solving; (iii) promoting healthy lifestyles and educating people about their conditions and how to self-manage; (iv) helping people to monitor their symptoms and know when to take appropriate action; (v) helping people to manage the social, emotional, and physical impacts of their conditions; and (vi) providing opportunities to share and learn from other service users [12,15]. Current research indicates that self-efficacy may mediate self-management skills, with programs based on self-efficacy being particularly effective in improving the psychological state and quality of life for individuals post-stroke [11]. Moreover, self-management programs grounded in behavior change theories, such as social cognitive theory, demonstrate a more substantial impact [16].

Self-efficacy, referring to an individual’s belief in their capability to learn and perform a specific behavior, is a central concept in social cognitive theory [17]. These beliefs can shape how individuals feel, think, motivate themselves, and behave regarding their health. Research suggests that when individuals with stroke are empowered to successfully manage their health issues, they are more likely to take charge, stay motivated, and demonstrate resilience in the face of challenges or setbacks [18,19,20]. Self-efficacy can act both as a mediator and an outcome, with studies showing its relationship to activity performance, participation, disability, mood, and quality of life after a stroke [19,21].

Considering previous findings and current evidence-informed interventions, a tailored approach was developed by adapting the Bridges Stroke Self-Management Program (Bridges SSMP) for the Portuguese context. The resulting program, ComVida (Bridges-PT), was created using a hybrid approach that involved Portuguese stroke survivors, informal caregivers, and health professionals through an iterative co-production process [22]. The prototyping stage results underscored the program’s potential to enhance functional capability and social participation among stroke survivors, addressing a significant gap in post-stroke care in Portugal. Specifically, the ComVida workbook and mobile app were evaluated for understandability, actionality, and usability, receiving high scores and a strong level of recommendation for use [22].

Therefore, this study aimed to evaluate the feasibility and implementation of the ComVida (Bridges-PT) program with stroke survivors in both acute health care and community settings in Portugal by assessing its impact on self-efficacy, physical function, health-related quality of life, and emotional state. Building on previous research, we hypothesized that the ComVida (Bridges-PT) self-management program would lead to significant improvements in self-efficacy, physical function, health-related quality of life, and emotional state among stroke survivors in Portugal over a 12-week period.

## 2. Materials and Methods

### 2.1. Research Design

A quasi-experimental study was conducted from October 2023 to July 2024, with a pre-test and post-test design without a comparison group. The main exposure was the ComVida (Bridges-PT), and the outcome variables were self-efficacy, physical function, health-related quality of life, and emotional state. Although the absence of a control group makes it challenging to attribute changes solely to the intervention, this study aimed to provide valuable insights that inform the design of future larger-scale studies.

### 2.2. Setting and Participants

The intervention was provided in two stroke units and a community rehabilitation clinic located in the district of Setúbal, Portugal. Individuals with a confirmed diagnosis of stroke, aged over 18 years, and able to read simple text or have an informal caregiver to assist were included in the study. Exclusion criteria included a clinical diagnosis of a severe mental or neuropsychiatric disorder that compromised their ability to participate in the study (e.g., severe depression with psychotic symptoms and/or marked suicidal ideation, schizophrenia, and other delusional disorders) and cognitive impairment (<24 in Mini Mental State Examination).

Since this was a feasibility study, we did not conduct a prospective sample size calculation. Given the exploratory nature of the study, our goal was to recruit 30 stroke participants across three sites, with the participation of six certified health professionals on the implementation of the Bridges self-management program over an 8-month recruitment period.

### 2.3. Intervention

Bridges SSMP is a complex intervention developed to support people with stroke in managing their condition and enhancing their independence [23,24]. Rooted in self-efficacy and behavior change principles, this program assists health professionals in integrating self-management core skills into their routine clinical practice. The intervention places emphasis on the language used during interactions with people with stroke (Table 1) and the effective use of self-management tools [25]. Bridges SSMP has been extensively implemented in the UK, Ireland, New Zealand, Sweden, and Estonia, demonstrating positive outcomes in terms of implementation, feasibility, and acceptability [23,24,25,26,27].

The ComVida (Bridges-PT) program was co-developed to empower people with stroke through a person-centered, self-management approach led by trained health professionals [22]. The term “ComVida” emerged from discussions held during co-creation workshops. Stroke survivors attributed this term to emphasize the importance of life after a stroke, metaphorically representing an invitation to life and hope. This concept underscores the focus on living fully and positively post-stroke, highlighting the importance of finding new life meaning, resilience, and optimism.

Three Portuguese teams of health professionals from hospital and community stroke rehabilitation received Bridges self-management training, accredited by the Personalised Care Institute, in eight virtual workshops over a six-month period. Bridges is a 5-stage program, co-produced and co-delivered with people with lived experience, aiming to lead to sustainable changes in culture and clinical practice.

The program sought to differentiate from standard stroke rehabilitation by personalized one-to-one rehabilitation sessions incorporating eleven principles to enhance self-management skills. Each therapy session is committed to inclusive self-management support that prioritizes the person’s story, emphasizes small achievements, and fosters supportive relationships, peer support and family/friends self-management skills and support, risk-taking, and self-reflection.

Moreover, the ComVida program combines in-person and digital approaches to support post-stroke self-management, including a stroke workbook and a mobile app [22]. The workbook fosters self-management by offering peer support and social learning, featuring experiences and recovery strategies from stroke survivors, and providing space for self-reflection, goal-setting, and tracking achievements. The mobile app, developed with a modular three-layer architecture, organizes information into a knowledge base data with FAQs and glossary and user-specific data like profiles, reminders, and diary entries. Despite the hybrid approach, this paper focuses on implementing the ComVida program with the stroke workbook. Findings related to the digital approach will be presented elsewhere.

### 2.4. Instruments

Clinical measures found sensitive in previous self-management trials and validated in stroke populations were utilized [23]. Additionally, a sociodemographic data form consisting of multiple-choice and fill-in-the-blank questions was used to cover gender, age, marital status, education level, professional status, and clinical characteristics (e.g., time after stroke, length of hospital stay, among others).

The Stroke Self-Efficacy Questionnaire (SSEQ) is a stroke-specific self-efficacy questionnaire designed to assess self-efficacy in individuals recovering from a stroke, particularly focusing on their degree of confidence in successfully performing tasks deemed significant [28]. The SSEQ is structured as a self-reported measure with 13 items, to assess self-efficacy in two main areas: self-management and functional performance. Psychometric evaluation has demonstrated its reliability and validity, with excellent internal consistency with Cronbach’s alfa of 0.90, a two-dimensional structure, and construct validity, with consistent correlations with other validated measures of health and functional independence [28,29]. Results from the validation process for Portugal showed a good fit of the data to the two-domain model, with a Cronbach’s alpha of 0.91 for the total scale, α = 0.91 for the activity subscale, and α = 0.80 for the self-management subscale [30].

The Stroke Impact Scale 16 (SIS-16) is a concise, stroke-specific instrument designed to assess physical function in stroke survivors [31]. The SIS-16 is derived from the SIS version 3.0 [32] and focuses on 16 items that measure physical domains, such as strength, hand function, mobility, and activities of daily living. Psychometric evaluations of the original SIS-16 and the Portuguese version have demonstrated their strong reliability and validity. Internal consistency is excellent, with Cronbach’s alpha values typically exceeding 0.90; test–retest reliability is also high, with intraclass correlation coefficients (ICCs) at approximately 0.95; and criterion validity is good, correlating well with other established measures of physical function [31,33].

The Hospital Anxiety and Depression Scale (HADS) is a self-assessment tool designed to detect anxiety and depression and consists of fourteen items divided into two subscales: seven items for anxiety (HADS-A) and seven for depression (HADS-D) [34]. Psychometric evaluations of the HADS have shown it to be a reliable and valid instrument, with high internal consistency for both subscales (Cronbach’s alpha values typically ranging from 0.68 to 0.932), strong test–retest reliability, with correlation coefficients at approximately 0.86 for the depression subscale and 0.89 for the anxiety subscale [35]. The Portuguese version showed metric properties similar to studies in other languages [36].

The 12-Item Short Form Health Survey version 2 (SF-12v2) is a brief, self-reported questionnaire designed to measure health-related quality of life across 12 questions that assess physical function, through the Physical Component Summary (PCS) and mental function with the Mental Component Summary (MCS). The PCS component covers physical function, physical performance, and general health, while the MCS component includes vitality, social function, emotional performance, and mental health [37]. Derived from the SF-36, the SF-12v2 covers the same eight health domains as the SF-36 with substantially fewer questions, making it a more practical research tool, especially among populations with limited attention spans or mental health problems. Psychometric evaluations of the SF-12v2 have demonstrated strong reliability and validity across different cultural contexts, including the Portuguese. Internal consistency is high, with Cronbach’s alpha values typically exceeding 0.80 for both the physical and mental health scores. Test–retest reliability is also robust, with intraclass correlation coefficients (ICCs) at approximately 0.89 and 0.86 for both. Construct validity is supported by significant correlations with other established measures of health status, such as the SF-36 and the EQ-5D2. Additionally, the SF-12v2 has shown good sensitivity to change, making it suitable for longitudinal studies assessing health outcomes over time [37,38,39,40].

### 2.5. Data Collection

**Figure 1:**
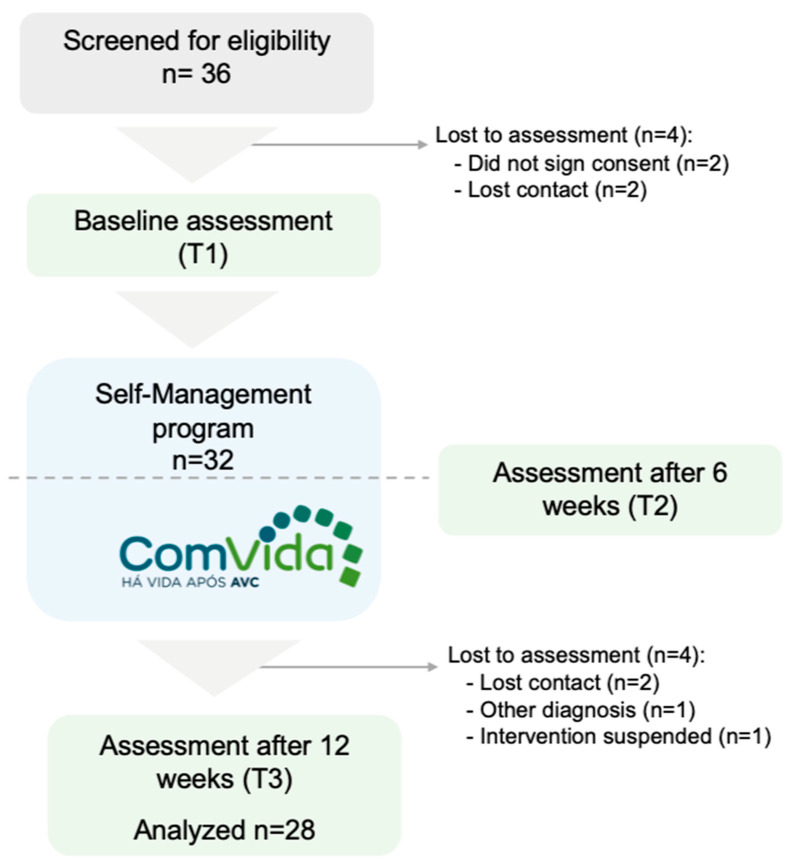
Study flow chart.

After assessing the eligibility of 36 potential participants for the study (as shown in Figure 1), 32 were assessed at baseline (T1), and 28 completed the intervention. The baseline assessment at T1 was conducted by an external researcher after obtaining the participant’s consent. This assessment included a socio-demographic questionnaire, the SSEQ, SIS-16, HADS, and SF-12v2. These assessments were repeated for all participants at the end of 6 weeks (T2) and 12 weeks (T3). The 12-week follow-up period was chosen to provide an initial assessment of the ComVida (Bridges-PT) program’s impact on self-efficacy, physical function, health-related quality of life, and emotional state. This follow-up period aligns with previous studies on the Bridges program, such as those by Jones et al. [23] and McKenna et al. [24], which demonstrated the feasibility and impact of a 12-week follow-up in stroke self-management programs.

### 2.6. Data Analysis

Data were analyzed using IBM SPSS Statistics software for Windows, Version 22.0. Descriptive statistics were computed to present means with standard deviation (SD) for continuous variables and percentages for categorical variables. After performing Shapiro–Wilk normality tests, paired sample T tests were conducted to determine the performance in self-efficacy, physical function, health-related quality of life, and emotional state after participation in the program. Pretest and post-test differences were considered statistically significant if *p* < 0.05. Clinical significance was determined by calculating the effect size using Cohen’s d. The effect size was considered small if d was between 0.2 and 0.5, medium if d was between 0.5 and 0.8, and large if d > 0.8. The Pearson correlation coefficient was also used to identify whether there was a correlation between the different evaluation moments.

### 2.7. Ethical Considerations

Ethical approval for this study was granted by the Ethics Commission of the “Unidade Local de Saúde Arrábida” (Reference n.16/2023F) and the “Unidade Local de Saúde do Litoral Alentejano” (n.018/2023). Participants were provided with an informational document detailing the study’s purpose, nature, and procedures at each stage of the project. This process was conducted in alignment with the ethical principles set forth by the World Medical Association Declaration of Helsinki. After addressing all questions, participants who consented to take part in the study provided written informed consent. Confidentiality and anonymity were maintained through a numerical coding system known only to the research team. Additionally, all data were securely stored and only accessible to authorized personnel, ensuring the protection of participants’ personal information throughout the study.

## 3. Results

The study included 28 participants (mean age: 66 ± 11.9 years) who experienced a stroke an average of 1.2 ± 1.5 months prior (Table 2). Of these, approximately 86% suffered an ischemic stroke, 75% (21 participants) were recruited from a hospital setting, and 25% (7 participants) from the community. The hospital group had a mean age of 65 ± 12.2 years and an average post-stroke duration of 0.5 ± 0.3 months. In contrast, the community group had a mean age of 70 ± 11.1 years and an average post-stroke duration of 3 ± 2.1 months.

The ComVida intervention was implemented by six healthcare professionals, including five physiotherapists and one nurse. The most frequently used principles focused on personalizing self-management support to the needs and personal circumstances, making it inclusive for everyone, prioritizing the people’s story and what is important for them, as well as on building supporting relationships and relinquishing control. On average, participants attended 7.2 (±6.9) sessions, with an average of four principles used per session (±3.12) and eight principles (±3.4) used throughout the entire intervention.

The outcomes at each time point (baseline, 6 weeks, and 12 weeks) are presented in Table 3.

### 3.1. Effect on Self-Efficacy

The results from SSEQ indicate a progressive increase in self-efficacy from baseline through twelve weeks (Table 3), with the mean score increasing by 10 points (10.04; *p* < 0.001) across the three time points. By the 12-week mark, the effect size of 1.57 is considered very large, indicating a strong impact of the intervention on self-efficacy. The *p*-value of less than 0.001 confirms that this result is highly statistically significant. The Pearson correlation coefficients provide additional insights into the relationships between the changes in self-efficacy and SSEQ subscales over time (Table 4), with the strong correlations for the SSEQ activity subscale at both 6 weeks and 12 weeks (0.618 and 0.697, respectively) indicating that improvements in self-efficacy related to activity were consistent and significant. The weaker correlations for the SSEQ self-management subscale indicate that, while there were improvements, they were not as consistent or significant as those for the SSEQ activity.

### 3.2. Effect on Physical Function

The results of SIS 16 indicate progressive and statistically significant improvements in physical function over the three assessment points (Table 3). The most significant gains occurred during the first 6 weeks, followed by additional improvements between 6 and 12 weeks. Cohen’s d values suggest a large effect size for all time points. The significant correlations in Table 4 suggest consistency in the pattern of change across participants. Specifically, the Pearson correlation coefficients indicate strong positive relationships between the different evaluation moments, meaning that improvements in physical function at one time point are strongly associated with improvements at subsequent time points.

### 3.3. Effect on Emotional State

Table 3 shows a steady significant decrease in HADS scores over time, with moderate to strong correlations indicating consistent patterns across the measurements. The correlations in Table 4 reveal that reductions in anxiety and depression scores at earlier time points are strongly related to further reductions at later time points. This suggests that participants who experienced improvements in their emotional state early in the program continued to show improvements over time.

### 3.4. Effect on Health-Related Quality of Life

The results on SF-12v2 (Table 3) show a significant increase in health-related quality of life over time, with consistency in the pattern of change across participants (Table 4) for both PCS and MCS scores of the scale. PCS scores showed significant improvement from baseline to 6 weeks and from baseline to 12 weeks, with large effect sizes. The correlations between different time points were moderate to strong, indicating consistent improvements over time. MCS scores also showed improvement, particularly from baseline to 12 weeks. The correlations between different time points were moderate to strong, suggesting consistent improvements in both physical and mental health-related quality of life over the study period.

## 4. Discussion

The study assessed the impact of a self-management program on stroke survivors within the Portuguese context, with results demonstrating significant improvements in self-efficacy, physical function, health-related quality of life, and emotional state.

Overall, there were significant differences in all evaluated outcomes, especially between six and twelve weeks. These findings are consistent with previous feasibility results of the Bridges program [23,24], which utilized a randomized controlled trial design in a community setting and employed mostly the same outcome measures, except for those assessing functional capability and quality of life. Despite these methodological differences, the consistently positive outcomes across studies highlight the potential of self-management programs to improve the well-being of stroke survivors. In particular, the more pronounced improvements observed during the last six weeks of our study may be explained by a gradual adaptation and increased use of self-management strategies. In the acute and subacute phases following a stroke, people tend to be more vulnerable. Over time, individuals seem to start adapting and integrating self-management principles, while receiving appropriate support [41].

In this study, self-efficacy emerged as a pivotal component of stroke self-management support. The moderate-to-large effect sizes indicate that participants’ confidence in managing their condition increased significantly, which is a critical factor for successful rehabilitation. The HADS results indicated significant reductions in anxiety and depression (e.g., at 12 weeks: d = −1.12) with medium to large effect sizes and moderate to strong correlations between different time points. Similarly, the SIS-16 findings demonstrated large effect size improvements in functional independence (e.g., at twelve weeks: d = 1.81). Given that 75% (N = 21) of the participants were recruited in a hospital setting, the support intervention focused on their transition to home, yielding results comparable to those reported by O’Callaghan et al. [42], which also demonstrated significant improvements in functional status at twelve weeks. According to these systematic and meta-analysis findings, similar improvements may be expected in reducing depression and anxiety at 6–12 months [42]. When compared with conventional rehabilitation (e.g., [43]), both approaches have shown beneficial effects on motor function, functional recovery, and quality of life. However, an integrated self-management approach has demonstrated additional benefits, including improvements in self-efficacy and a reduction in anxiety and depression levels.

These results underscore the mediating role of self-efficacy in facilitating positive emotional and functional outcomes, in alignment with self-efficacy theory [44]. Similar programs underpinned by Bandura’s construct of self-efficacy (e.g., [45]) and narrative therapy approach, including group sessions [46], reported moderate improvements in self-efficacy and associated emotional and functional outcomes. The very large effect size for 12 weeks in the SSEQ suggests that the participants’ self-efficacy may have outperformed others in fostering self-efficacy over time. However, looking at the results of the subscales SSEQ activity and SSEQ self-management, only the first indicated significant improvements, which may indicate the need for time to rediscover problem-solving abilities [47].

In comparison to other studies of self-management in stroke populations, this study was limited by a small sample size. While the sample size may limit the statistical power to detect smaller effect sizes, it allowed for the identification of significant trends. The exclusion of stroke survivors with severe language and cognitive impairments and the lack of differentiation among participants based on stroke type and level of physical independence were also a limitation of the study. Moreover, there was no control group and consequent randomization, with a risk of potential bias. Additionally, a follow-up period of 6 to 12 months would be beneficial to assess the long-term effectiveness of the program. While the study timeframe allowed for the identification of significant short-term trends, it remains undetermined whether the observed improvements continue to progress over an extended period.

While our results demonstrate significant improvements in self-efficacy, physical function, health-related quality of life, and emotional state among stroke survivors in Portugal, several factors should be considered when applying these findings to other populations. The generalizability of findings to other age groups, geographic regions, and healthcare settings may be limited. Future studies should include more diverse samples to enhance the representativeness of the findings. Moreover, the ComVida (Bridges-PT) program was tailored to the Portuguese context, incorporating cultural and linguistic adaptations. While the core principles of the Bridges program are universally applicable, cultural differences may influence the program’s effectiveness in other settings. Replicating this study in different cultural contexts would provide valuable insights into the program’s adaptability and generalizability. The intervention was delivered by trained health professionals, who received specific training in the Bridges self-management approach. The availability and training of healthcare providers may vary across different regions and healthcare systems, potentially affecting the program’s implementation and outcomes. Future research should explore the feasibility and effectiveness of the program in diverse healthcare settings.

Our quasi-experimental design, while informative, lacks the rigor of randomized controlled trials (RCTs). The absence of a control group limits the ability to attribute changes solely to the intervention. Future research should incorporate RCT designs to strengthen the evidence base and enhance the generalizability of the findings.

## 5. Conclusions

This study evaluated the impact of the ComVida (Bridges-PT) self-management program on stroke survivors in Portugal, demonstrating significant improvements in self-efficacy, physical function, health-related quality of life, and emotional state over a 12-week period. The findings are consistent with previous research on the Bridges program, highlighting its potential as a valuable component of post-stroke care. Despite methodological differences and limitations, such as the quasi-experimental design and specific sample characteristics, the positive outcomes underscore the program’s effectiveness.

Future research should focus on replicating these findings in larger, more diverse populations and different cultural contexts to enhance the generalizability of the results. Incorporating randomized controlled trial designs and exploring long-term effects beyond 12 weeks would provide more robust evidence of the impact of the program. Additionally, investigating the integration of digital tools, such as mobile apps, alongside traditional self-management approaches could offer new insights into optimizing stroke rehabilitation.

## Figures and Tables

**Table 1:** Core self-management skills in Bridges/ComVida self-management program.

**Reflection:**	Supporting people to reflect on their progress and useful strategies, helping them to attribute changes and improvements to personal effort, not the skills of the health professional.
**Problem-solving:**	Supporting people to think through problems together and come up with different ideas, strategies, and ways to adjust, rather than relying on suggestions from health professionals.
**Self-discovery:**	Supporting people to try new ways of doing things and try out different activities and strategies, which may involve taking risks.
**Goal setting:**	Avoiding clinician-led goals, focusing on patient priorities, and what is meaningful and relevant. Encouraging small steps to promote feelings of success and working towards longer-term aspirational goals.
**Taking action:**	Supporting people to do more, even small things, and appraising their efforts.
**Support:**	Supporting people to access their support network, and available resources in the community.
**Knowledge:**	Supporting people to develop greater self-awareness about what works for their own situation and challenges, and giving them meaningful information.

**Table 2:** Sociodemographic and clinical characteristics of the participants.

Demographic Characteristics	N	%	Mean	SD	Min–Max
Gender					
Female	19	67.9			
Male	9	32.1			
Age (years)			66.4	12	34–86
Marital status					
Single	4	14.3			
Married	20	71.4			
Divorced	3	10.7			
Widow	1	3.6			
Educational level					
Primary school	11	39.3			
High school diploma	11	39.3			
University degree	6	21.4			
Professional status					
Unemployed	3	10.7			
Retired	17	60.7			
Medical leave/unable to work	8	28.6			
Recruitment context					
Hospital	21	75			
Community	7	25			
Type of stroke					
Ischemic	24	85.7			
Hemorrhagic	4	14.3			
Time after stroke (months)			1.2	1.5	0.1–5
Length of hospital stay (days)			19.9	18.3	2–90
In-patient rehabilitation (yes)	8	28.4			
Length of rehabilitation stay (months)			1.3	1.4	0.23–4

**Table 3:** Means, SDs at all time points and outcomes analysis.

Outcome	Baseline Mean (SD)	6 Weeks Mean (SD)	12 Weeks Mean (SD)	Effect Size (6 Weeks)	Effect Size (12 Weeks)
Self-Efficacy (SSEQ Total)	23.3 (7.7)	29.6 (10.5)	33.3 (6.1)	0.67 (*p* < 0.001)	1.57 (*p* < 0.001)
Physical Function (SIS-16)	47.5 (12.2)	61.2 (13.6)	67.2 (12.9)	1.31 (*p* < 0.001)	1.81 (*p* < 0.001)
Emotional State (HADS Total)	12.6 (6.9)	8.6 (5.9)	5.9 (5.2)	−0.64 (*p* = 0.002)	−1.12 (*p* < 0.001)
Quality of Life					
SF-12v2—PCS	31.9 (4.9)	34.9 (6.5)	35.2 (6.01)	1.047 (*p* = 0.019)	1.58 (*p* = 0.012)
SF-12v2—MCS	41.9 (5.7)	41.9 (6.12)	44.4 (6.7)	0.96 (*p* = 0.096)	1.49 (*p* = 0.018)

SSEQ—Stroke Self-Efficacy Scale; SIS-16—Stroke Impact Scale 16; SF-12v2—12 item Short Form Health Survey version 2; PCS—Physical Component Summary; MCS—Mental Component Summary.

**Table 4:** Correlations between time points.

	Total		Subscales			
			Activity		Self-Management	
	Pearson	*p*-Value	Pearson	*p*-Value	Pearson	*p*-Value
SSEQ Total						
Difference at 6 weeks	0.481 **	0.010	0.618 **	<0.001	0.084	0.671
Difference 6–12 weeks	0.845 **	<0.001	0.875 **	<0.001	0.35	0.068
Difference at 12 weeks	0.592 **	<0.001	0.697 **	<0.001	0.282	0.146
SIS16						
Difference at 6 weeks	0.677 **	<0.001				
Difference 6–12 weeks	0.898 **	<0.001				
Difference at 12 weeks	0.627 **	<0.001				
HADS			HADS-A		HADS-D	
Difference at 6 weeks			0.674 **	<0.001	0.481 **	0.01
Difference 6–12 weeks			0.725 **	<0.001	0.635 **	<0.001
Difference at 12 weeks			0.615 **	<0.001	0.414 *	0.029
SF-12v2			PCS		MCS	
Difference at 6 weeks			0.619 **	<0.001	0.625 **	<0.001
Difference 6–12 weeks			0.587 *	0.001	0.616 **	<0.001
Difference at 12 weeks			0.446 *	0.017	0.468 *	0.012

SSEQ—Stroke Self-Efficacy Scale; SIS16—Stroke Impact Scale 16 item; HADS—Hospital Anxiety and Depression Scale; HADS-A, Hospital Anxiety and Depression Scale—Anxiety scores; HADS-D, Hospital Anxiety and Depression Scale—Depression scores; SF-12v2—12 item Short Form Health Survey version 2; PCS—Physical Component Summary; MCS—Mental Component Summary. *p* = Significance Level; * *p* < 0.05; ** *p* < 0.001.

## Data Availability

The original contributions presented in this study are included in the article. Further inquiries can be directed to the corresponding author.

## References

[1] Taylor S.J., Pinnock H., Epiphaniou E., Pearce G., Parke H.L., Schwappach A., Purushotham N., Jacob S., Griffiths C.J., Greenhalgh T. (2014). A rapid synthesis of the evidence on interventions supporting self-management for people with long-term conditions: PRISMS—Practical systematic RevIew of Self-Management Support for long-term conditions. Health Soc. Care Deliv. Res..

[2] Audulv Å., Hutchinson S., Warner G., Kephart G., Versnel J., Packer T.L. (2021). Managing everyday life: Self-management strategies people use to live well with neurological conditions. Patient Educ. Couns..

[3] NICE (2023). National Clinical Guidelines for Stroke for the United Kingdom and Ireland.

[4] Feigin V.L., Stark B.A., Johnson C.O., Roth G.A., Bisignano C., Abady G.G., Abbasifard M., Abbasi-Kangevari M., Abd-Allah F., Abedi V. (2021). Global, regional, and national burden of stroke and its risk factors, 1990–2019: A systematic analysis for the Global Burden of Disease Study 2019. Lancet Neurol..

[5] GBD 2021 Diseases and Injuries Collaborators (2024). Global incidence, prevalence, years lived with disability (YLDs), disability-adjusted life-years (DALYs), and healthy life expectancy (HALE) for 371 diseases and injuries in 204 countries and territories and 811 subnational locations, 1990–2021: A system. Lancet.

[6] DGS (2021). Plano Nacional de Saúde 2021–2030. Saúde Sustentável: De Tod@s para Tod@s [National Health Plan 2021–2030. Sustainable Health: From Everyone for Everyone].

[7] Abbafati C., Machado D.B., Cislaghi B., Salman O.M., Karanikolos M., McKee M., Abbas K.M., Brady O.J., Larson H.J., Trias-Llimós S. (2020). Global burden of 369 diseases and injuries in 204 countries and territories, 1990–2019: A systematic analysis for the Global Burden of Disease Study 2019. Lancet.

[8] Winstein C.J., Stein J., Arena R., Bates B., Cherney L.R., Cramer S.C., Deruyter F., Eng J.J., Fisher B., Harvey R.L. (2016). Guidelines for Adult Stroke Rehabilitation and Recovery: A Guideline for Healthcare Professionals from the American Heart Association/American Stroke Association. Stroke.

[9] Mountain A., Patrice Lindsay M., Teasell R., Salbach N.M., de Jong A., Foley N., Bhogal S., Bains N., Bowes R., Cheung D. (2020). Canadian Stroke Best Practice Recommendations: Rehabilitation, Recovery, and Community Participation following Stroke. Part Two: Transitions and Community Participation Following Stroke. Int. J. Stroke.

[10] Feigin V.L., Owolabi M.O., Feigin V.L., Abd-Allah F., Akinyemi R.O., Bhattacharjee N.V., Brainin M., Cao J., Caso V., Dalton B. (2023). Pragmatic solutions to reduce the global burden of stroke: A World Stroke Organization–Lancet Neurology Commission. Lancet Neurol..

[11] Fryer C.E., Luker J.A., McDonnell M.N., Hillier S.L. (2016). Self-Management Programs for Quality of Life in People with Stroke. Stroke.

[12] Warner G., Packer T., Villeneuve M., Audulv A., Versnel J. (2015). A systematic review of the effectiveness of stroke self-management programs for improving function and participation outcomes: Self-management programs for stroke survivors. Disabil. Rehabil..

[13] Parke H.L., Epiphaniou E., Pearce G., Taylor S.J.C., Sheikh A., Griffiths C.J., Greenhalgh T., Pinnock H. (2015). Self-management support interventions for stroke survivors: A systematic meta-review. PLoS ONE.

[14] Prados-Román E., Cabrera-Martos I., Martín-Nuñez J., Valenza-Peña G., Granados-Santiago M., Valenza M.C. (2024). Effectiveness of self-management interventions during the peri-hospitalization period in patients with stroke: A systematic review and meta-analysis. Clin. Rehabil..

[15] Murphy M. (2000). Helping people help themselves. J. AWWA.

[16] Lau S.C.L., Judycki S., Mix M., DePaul O., Tomazin R., Hardi A., Wong A.W.K., Baum C. (2022). Theory-Based Self-Management Interventions for Community-Dwelling Stroke Survivors: A Systematic Review and Meta-Analysis. Am. J. Occup. Ther..

[17] Bandura A. (1977). Self-efficacy: Toward a unifying theory of behavioral change. Psychol. Rev..

[18] Gangwani R., Cain A., Collins A., Cassidy J.M. (2022). Leveraging Factors of Self-Efficacy and Motivation to Optimize Stroke Recovery. Front. Neurol..

[19] Lo S.H.S., Chau J.P.C., Lam S.K.Y., Saran R., Choi K.C., Zhao J., Thompson D.R. (2022). Association between participation self-efficacy and participation in stroke survivors. BMC Neurol..

[20] Szczepańska-Gieracha J., Mazurek J. (2020). The role of self-efficacy in the recovery process of stroke survivors. Psychol. Res. Behav. Manag..

[21] Amiri F.S., Abolhassani S., Alimohammadi N., Roghani T. (2022). Investigating the effect of self-management program on stroke’s patients’ self-efficacy. BMC Neurol..

[22] Pereira C.M., Matos M., Carvalho D., Macedo P., Calheiros J.M., Alves J., Ferreira L.P., Dias T.L., Madeira R.N., Jones F. (2024). Building Bridges between People with Stroke, Families, and Health Professionals: Development of a Blended Care Program for Self-Management. J. Clin. Med..

[23] Jones F., Gage H., Drummond A., Bhalla A., Grant R., Lennon S., McKevitt C., Riazi A., Liston M. (2016). Feasibility study of an integrated stroke self-management programme: A cluster-randomised controlled trial. BMJ Open.

[24] McKenna S., Jones F., Glenfield P., Lennon S. (2015). Bridges self-management program for people with stroke in the community: A feasibility randomized controlled trial. Int. J. Stroke.

[25] Hale L., Jones F., Mulligan H., Levack W., Smith C., Claydon L., Milosavljevic S., Taylor D., Allan J., MacKenzie N. (2014). Developing the bridges self-management programme for New Zealand stroke survivors: A case study. Int. J. Ther. Rehabil..

[26] Hale L., McCulloch M., De Ruiter S., Wihongia E., Norlinga E.M., Gorczynski D., Kennedy P., Jones F. (2021). Implementing and evaluating the bridges stroke self-management programme into a new zealand district health board stroke service: A case study. N. Z. J. Physiother..

[27] Singer B., Jones F., Lennon S., Singer B. (2018). Adapting the Bridges stroke self-management programme for use in Australia. Int. J. Ther. Rehabil..

[28] Jones F., Partridge C., Reid F. (2008). The Stroke Self-Efficacy Questionnaire: Measuring individual confidence in functional performance after stroke. J. Clin. Nurs..

[29] Riazi A., Aspden T., Jones F. (2014). Stroke self-efifcacy questionnaire: A Rasch-refined measure of confidence post stroke. J. Rehabil. Med..

[30] Figueira C. (2023). Adaptação Cultural e Contributo Para a Validação do Stroke Self-Efficacy Questionnaire (SSEQ) [Cultural Adaptation and Contribution to the Validation of the Portuguese Version of the Stroke Self-Efficacy Questionnaire (SSEQ)].

[31] Duncan P.W., Lai S.M., Bode R.K., Perera S., DeRosa J. (2003). Stroke impact scale-16: A brief assessment of physical function. Neurology.

[32] Duncan P.W., Bode R.K., Lai S.M., Perera S. (2003). Rasch analysis of a new stroke-specific outcome scale: The stroke impact scale. Arch. Phys. Med. Rehabil..

[33] Teodoro M., Pereira C.M. (2009). Contributo Para a Adaptação e Validação da Versão Portuguesa do Questionário de Impacto do AVC-Versão 3.0 e Versão Proxy [Contribution to the Adaptation and Validation of the Portuguese Version of the Stroke Impact Questionnaire—Version 3.0 and Proxy].

[34] Zigmond A.S., Snaith R.P. (1983). The Hospital Anxiety and Depression Scale. Acta Psychiatr. Scand..

[35] Bjelland I., Dahl A.A., Haug T.T., Neckelmann D. (2002). The validity of the Hospital Anxiety and Depression Scale: An updated literature review Ingvar. J. Psychosom. Res..

[36] Pais-Ribeiro J., Silva I., Ferreira T., Martins A., Meneses R., Baltar M. (2007). Validation study of a Portuguese version of the Hospital Anxiety and Depression Scale. Psychol. Health Med..

[37] Ware J.E., Kosinski M., Keller S.D. (1996). A 12-Item Short-Form Health Survey: Construction of Scales and Preliminary Tests of Reliability and Validity. Med. Care.

[38] Pais-Ribeiro J.L. (2005). O importante é a Saúde: Estudo de Adaptação de um Instrumento para Avaliar o Estado de Saúde.

[39] Soh S.E., Morello R., Ayton D., Ahern S., Scarborough R., Zammit C., Brand M., Stirling R.G., Zalcberg J. (2021). Measurement properties of the 12-item Short Form Health Survey version 2 in Australians with lung cancer: A Rasch analysis. Health Qual. Life Outcomes.

[40] Huo T., Guo Y., Shenkman E., Muller K. (2018). Assessing the reliability of the short form 12 (SF-12) health survey in adults with mental health conditions: A report from the wellness incentive and navigation (WIN) study. Health Qual. Life Outcomes.

[41] Rutherford S.J., Hocking C., Theadom A., McPherson K.M. (2018). Exploring challenges at 6 months after stroke: What is important to patients for self-management?. Int. J. Ther. Rehabil..

[42] O’Callaghan G., Fahy M., Murphy P., Langhorne P., Galvin R., Horgan F. (2022). Effectiveness of interventions to support the transition home after acute stroke: A systematic review and meta-analysis. BMC Health Serv. Res..

[43] Roesner K., Scheffler B., Kaehler M., Schmidt-Maciejewski B., Boettger T., Saal S. (2024). Effects of physical therapy modalities for motor function, functional recovery, and post-stroke complications in patients with severe stroke: A systematic review update. Syst. Rev..

[44] Bandura A. (2004). Health promotion by social cognitive means. Health Educ. Behav..

[45] Lo S.H.S., Chang A.M., Chau J.P.C. (2018). Stroke self-management support improves survivors’ self-efficacy and outcome expectation of self-management behaviors. Stroke.

[46] Johnson V.L., Apps L., Kreit E., Mullis R., Mant J., Davies M.J. (2023). The feasibility of a self-management programme (My Life After Stroke; MLAS) for stroke survivors. Disabil. Rehabil..

[47] Nott M., Wiseman L., Seymour T., Pike S., Cuming T., Wall G. (2021). Stroke self-management and the role of self-efficacy. Disabil. Rehabil..

